# SOCIAL ISOLATION AND COGNITIVE IMPAIRMENT IN OLDER ADULTS WITH DIABETES

**DOI:** 10.1093/geroni/igad104.2734

**Published:** 2023-12-21

**Authors:** Bohyun Kim, Jie Hu

**Affiliations:** The Ohio State University, Columbus, Ohio, United States; The Ohio State University, Columbus, Ohio, United States

## Abstract

Social isolation is a notable influencing factor contributing to cognitive impairment in older adults with diabetes, leading to an increased risk of dementia. As the specific mechanisms by which social isolation affect cognitive health have not been fully understood, it is important to identify the underlying mechanisms. This study aims to examine the pathways between social isolation and the psychological mediators of cognitive impairment in older adults with type 2 diabetes. A secondary data analysis was conducted using the National Social Life, Health, and Aging Project Wave 3 Database (2015-2016) of 725 older adults with diabetes aged 60 and over. The Social Disconnectedness Scale, Perceived Isolation Scale, Center for Epidemiological Studies Depression Scale, Hospital Anxiety and Depression Scale, and Perceived Stress Scale were used. Structural equation modeling was employed to investigate the complex interplay of social isolation and psychological mediators with cognitive impairment. Bootstrapping procedures were used to assess significance of indirect effects through psychological mediators. The hypothesized model was supported with adequate fit to the data (CFI=0.90; RMSEA=0.05; SRMR=0.08). Social disconnectedness had an indirect effect on cognitive health through perceived isolation and perceived stress (γ= -0.122, p< 0.01). However, there was no significant indirect effect through anxiety and depressive symptoms. The model provides a framework for developing interventions to reduce cognitive impairment in socially isolated older adults with diabetes. Understanding the pathways between social isolation and cognitive impairment will provide a foundation for controlling modifiable factors to prevent the progression of dementia in older adults with diabetes.

